# Changes in Standing Postural Control Ability in a Case of Spinocerebellar Ataxia Type 31 With Physical Therapy Focusing on the Center of Gravity Sway Variables and Lower Leg Muscle Activity

**DOI:** 10.7759/cureus.51033

**Published:** 2023-12-24

**Authors:** Shiori Iwasa, Ryo Akaguchi, Hiroyuki Okuno, Koji Nakanishi, Kozo Ueta, Shu Morioka

**Affiliations:** 1 Physical Therapy, Faculty of Health Sciences, Kio University, Nara, JPN; 2 Neurorehabilitation, Graduate School of Health Sciences, Kio University, Nara, JPN; 3 Rehabilitation, Setsunan General Hospital, Osaka, JPN; 4 Physical Medicine and Rehabilitation, Neurorehabilitation Research Center, Kio University, Nara, JPN

**Keywords:** cerebellar ataxia, spinocerebellar degeneration, spinocerebellar ataxia, rehabilitation, postural balance control training

## Abstract

Spinocerebellar degeneration (SCD) is a progressive disease characterized by cerebellar ataxia or the posterior spinal cord. Among these, spinocerebellar ataxia type 31 (SCA31) is genetically more common in the Japanese population and is characterized by pure ataxia, resulting in severe disturbances in postural balance, with common falls. Therefore, rehabilitation is important to improve postural balance. Light touch is a known method of reducing postural sway, which acts with the light touching of an object with the body. We herein present a case of a patient with SCA31 who was trained in a standing position by lightly touching the back of the body to a wall surface. Dynamic interarticular coordination exercises were also performed as part of the rehabilitation program. As a result, even in the progressive SCA31, improvements in standing postural balance and activities of daily living contributed to improvements in the patient's postural balance. We followed the progress of postural control ability using the center of gravity sway measurement and electromyography and described some interesting characteristics of the patient's postural control ability in this report.

## Introduction

Spinocerebellar degeneration (SCD) describes progressive disorders with ataxia, caused by neurodegeneration of the cerebellum or posterior spinal cord, as the primary symptom. SCD can be solitary, such as multiple system atrophy (MSA), or hereditary, such as spinocerebellar ataxia (SCA). SCA is also known as autosomal dominant SCD, and 35 causative genes or loci have been identified to date [1]. Spinocerebellar ataxia type 31 (SCA31) is genetically more common in the Japanese population [2] and is characterized by an older age of onset compared to other types of SCA, slow progression, and pure cerebellar ataxia [3]. Limb ataxia, deep sensory deficits, and psychoneurotic signs are commonly presented in SCA31 [4].

In addition to ataxia, deep sensory deficits hinder the maintenance of postural balance in SCD. Indeed, impairment in postural balance and gait are the main features of SCD, leading to falls in many patients [5-7]. Hence, providing rehabilitation that focuses on improving the postural balance of patients with SCD is essential to prevent falls. Patients with SCD have impaired motor control coordination between joints because of ataxia [8]. The loss of coordinated movement results in co-contraction of the lower extremity muscles, leading to compensatory strategies such as increased joint stiffness to maintain standing postural balance [9,10]. Intentional stiffness strategies increase sway velocity and frequency in the postural sway index [11]. Moreover, intentionally reducing sway increases co-contraction of the lower leg muscles [12]. Postural control based on ankle muscle hyperactivity is referred to as a stiffness strategy [13]. Depending on their disease stage and the absence of neuropsychiatric symptoms, patients with SCA tend to adopt an intentional stiffness strategy.

Light touch is a well-known method for reducing postural sway [14]. In recent years, systematic reviews have shown that lightly touching an object with the fingertips and employing additional somatosensory information are effective tools to reduce postural sway in patients with postural balance disorders [15]. In principle, the parts of the body used for the light-touch effect are the fingertips, which are free of sensory disorders. However, patients with SCA-induced limb ataxia may not receive sufficient sensory information from their fingertips. The effects of light touch have only been studied in one patient with a progressive disease, namely, Parkinson's disease [16], and such a method is not currently indicated for SCA. Research on patients with peripheral neurosensory dysfunction and the elderly has shown that the light touch effect is more effective with the application of the sensation to the upper part of the body [17]. Additionally, although the study employed interpersonal touch rather than lightly touching an object, the more points of contact, the better the postural stability effect in patients with chronic stroke [18]. Herein, we report a case in which we adapted the task of maintaining a relaxed standing posture by lightly touching the back of the body to a wall as a physiotherapy intervention for a patient with SCA. When the patient's static standing postural balance stabilized to a reasonable degree, dynamic interarticular coordination exercises were initiated. We observed an improvement in the patient's standing postural balance, which contributed to the amelioration of ataxia and activities of daily living (ADL).

## Case presentation

Case introduction

**Figure 1:**
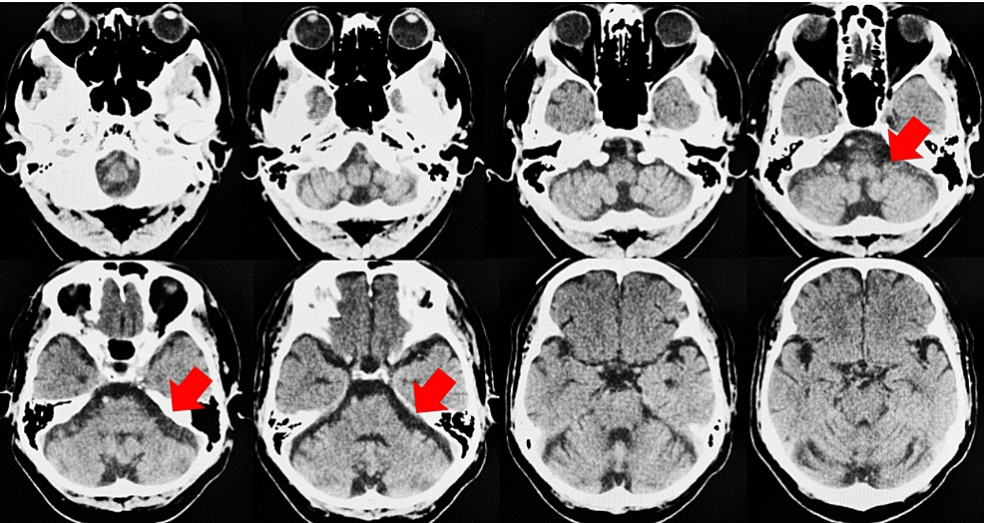
Computed tomography imaging Computed tomography imaging of a 60-year-old male patient with spinocerebellar ataxia type 31 (SCA31), sectioned in the frontal plane. The culmen exhibits prominent atrophy, and the declive, folium, and tuber vermis appear atrophic. Cerebellar atrophy is observed only in the lobulus quadrangularis.

**Figure 2:**
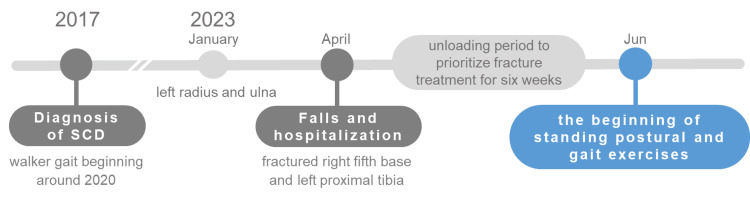
Timeline from the diagnosis of SCD Timeline from the diagnosis of SCD to the beginning of standing postural and gait exercises after a fall and hospitalization. SCD: spinocerebellar degeneration.

A 60-year-old man had been diagnosed with SCA31 six years prior to our intervention, with computed tomography (CT) showing cerebellar atrophy (Figure 1). SCD was diagnosed in 2017, and he has ambulated with the aid of a walker since approximately 2020. In January 2023, the patient suffered a forward fall and presented with left radius and ulnar fractures. In April of the same year, fractures in the base of the right fifth metatarsal and left proximal tibia were diagnosed after a second fall, and he was admitted to our hospital for rehabilitation. In June, the patient began bearing weight in the standing position (Figure 2). A comprehensive evaluation was performed in the initial phase of standing and walking exercises, including an assessment of postural balance-related information, finding muscle strength in the initial phase of standing and walking exercises of 4-5 (normal, 5) in the lower extremities on the Manual Muscle Test (MMT), indicating little to none muscle weakness. The tactile sensations in the extremities and trunk were unaltered, yet the deep vibration sensation in the extremities was slightly reduced, with normal movement. His Scale for the Assessment and Rating of Ataxia (SARA) score was 17.5/40, with 2/4 right and 3/4 left on the nose-finger test, and 1/4 right and 3/4 left on the finger test, indicating upper extremity ataxia. The heel-to-toe test scores were 4/4, indicating pronounced lower extremity ataxia, as well as reduced functionality in the gait (5/8) and stance (3/6) measures, reflecting a decline in both standing and walking scores. The total score of 17.5/40 indicated marked motor ataxia (Table 1), with ataxia being more pronounced in the left upper and lower limbs. The SARA consists of eight items with a total score ranging from 0 (no ataxia) to 40 points, indicating the severity of ataxia. The following items are assessed: (1) gait (0-8 points); (2) stance (0-6 points); (3) sitting (0-4 points); (4) speech disturbance (0-4 points); (5) finger chase (0-4 points); (6) nose-finger test (0-4 points); (7) rapid alternating hand movements (0-4 points); and (8) heel-shin slide (0-4 points). For items (5)-(8) of the SARA test for limb ataxia, both sides are scored, and the average score is calculated and used as the total score. Additionally, the patient presented a score of 23/56 on the Berg Balance Scale (BBS), a comprehensive postural balance test, characterizing an impaired standing postural balance. All the tests were performed by a physical therapist.

**Table 1:** Scale for the Assessment and Rating of Ataxia (SARA) Initial, six weeks after fracture; middle, seven weeks after fracture; late, eight weeks after fracture; final, nine weeks after fracture.

	Initial	Middle	Late	Final
1: Gait	5	5	4	4
2: Standing	3	3	2	2
3: Sitting	0	0	0	0
4: Speech impairment	2	2	2	2
5: Finger tracking test	1	1	1	1
6: Finger nose test	1	1	1	1
7: Hand turning in and out movements	1.5	1.5	1.5	1.5
8: Heel-shin test	4	4	3.5	3.5
Total:	17.5	17.5	15	15

Physical therapy intervention

**Figure 3:**
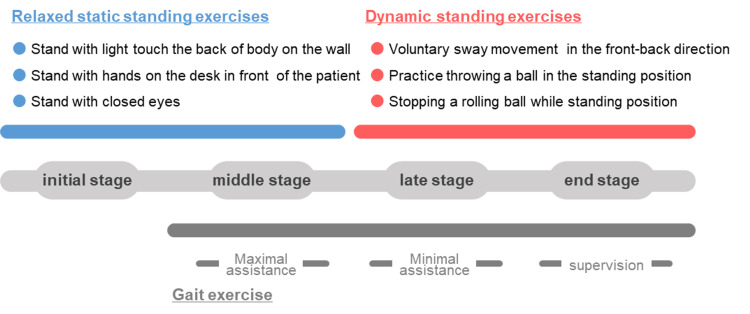
Physiotherapy exercises Initial stage, six weeks after fracture; middle stage, seven weeks after fracture; late stage, eight weeks after fracture; final stage, nine weeks after fracture. Physiotherapy exercises primarily aimed to improve standing postural balance over two periods, initial to middle and late to final (at discharge) stages. The examination and measurement at four time points, divided into initial, middle, late, and final (at discharge). A one-week interval is present between each stage.

Physical therapy sessions were organized into four weekly phases, i.e., initial, mid, late, and final (at discharge), with a primary focus on improving standing postural balance (Figure 3). During the initial to mid phases, we performed static postural balance exercises, which included tasks such as (1) maintaining a standing position with light contact of the back (cervical spine) against a wall, (2) maintaining a standing position with the hands placed on a desk in front of the body, and (3) maintaining a standing position with the eyes closed. The patients were instructed to "relax and maintain a standing position" for all tasks. Notably, the level of difficulty increased in an ascending fashion from tasks (1) to (3), with such being performed in a staged manner. The purpose of the exercises was to stabilize fluctuations in static standing control as much as possible, prevent excessive contraction of the lower limb muscles, and emphasize somatosensory perception. In the final phase, the following tasks were performed: (4) voluntary anterior-posterior center of gravity shift; (5) throwing a cue ball while standing; and (6) stopping a rolling ball with the lower limbs while standing. Similar to the static postural balance exercises, the level of difficulty increased in the ascending sequence from (4) to (6). The purpose of the tasks was to induce voluntary postural sway during dynamic postural control, coordinate joint movements, and improve inter-articular coordination throughout the body. Walking exercises were performed simultaneously, as shown in Figure 3.

Evaluation and statics

Assessments and measurements were performed at four time points (Figure 3). Daily living mobility was categorized as follows: not possible, wheelchair assistance, wheelchair supervision, wheelchair self-propulsion, walker assistance, walker supervision, and walker independence. Whereas the walking ability was categorized as: not possible, heavy assistance, moderate assistance, light assistance, watchful waiting, indoor independence, and outdoor independence. The degree of ataxia was assessed using the SARA scale and postural balance was assessed using the BBS. A licensed physical therapist administered the comprehensive assessment battery.

A BASYS center of pressure (CoP) sway meter (Tech Gihan, Kyoto, Japan) was used to measure postural balance, and the patients were instructed to maintain a standing position for 30 seconds. During the measurement, patients were instructed to place both upper limbs on the outside of their torso and focus on a marker placed 2 meters in front of them. Data were recorded for 30 seconds at a sampling rate of 1000 Hz. The CoP data obtained from the sway meter were processed using a 4th-order Butterworth filter with a low-pass cutoff frequency of 10 Hz. The following parameters were derived from the CoP data and used as indicators: the 95% confidence ellipse area (cm2), referred to as the sway area; the mean velocity (cm/s), referred to as the sway velocity; and a power spectrum analysis, used to classify the frequency band into low frequency (LF; 0.15-0.3 Hz), mid frequency (MF; 0.3-1 Hz), and high frequency (HF; 1-3 Hz), with the average power values calculated for each. We used the MuscleBIT surface electromyography (EMG) system (Plux, Lisbon, Portugal), which is capable of recording four-channel data via Bluetooth wireless transmission, to measure lower leg muscle activity. The measurement was synchronized with the aforementioned CoP sway meter. Surface electrodes were placed on both the tibialis anterior (TA) and soleus (SOL) muscles with a minimum distance of 10 cm to minimize crosstalk [14]. Electrodes were applied to the skin after thorough preparation with alcohol swabs to reduce impedance and securely attached using tape to ensure that the relative position between the electrodes and the subcutaneous muscles remained constant. The sampling frequency was set at 1000 Hz, consistent with the CoP measurements, and the signal was band-pass filtered and rectified to calculate the average amplitude (mV/s) during maintenance of the standing position. The co-contraction index (CI) between the TA and SOL was determined from the normalized EMG waveforms by calculating the overlap rate of the TA and SOL using the following formula [15,16]:

$I_a=\int_{t1}^{t2} EMG_{agon} (t)dt+\int_{t2}^{t3}EMG_{ant} (t)dt$

$I_{total}=\int_{t1}^{t3}[ EMG_{agon} +EMG_{ant} ](t)dt$

$CI=\frac{2I_a}{I_{total}} \times 100$

In addition, cross-lagged time-lag correlation analyses were performed at four time points (initial, mid, late, and final), with SARA and BBS scores as dependent variables and the CI of the left and right TA-SOL muscles as independent variables. Cross-lagged time-lag correlation analysis is a statistical method used to analyze the temporal order of relationships between variables. The analysis was performed using SMA version 07.30.20 (Simulation Modeling Analysis Program for Short Streams of Time-Series Data). SARA and the CI were analyzed using non-parametric methods with the Bonferroni correction. The significance level was set at 5%. In addition, data on sway area and velocity, mean power values in different frequency bands, lower leg muscle activity, and CI were obtained from a healthy, age-matched male subject in his 60s. These data were used as references for comparison.

Results of the intervention

ADL Progress

**Figure 4:**
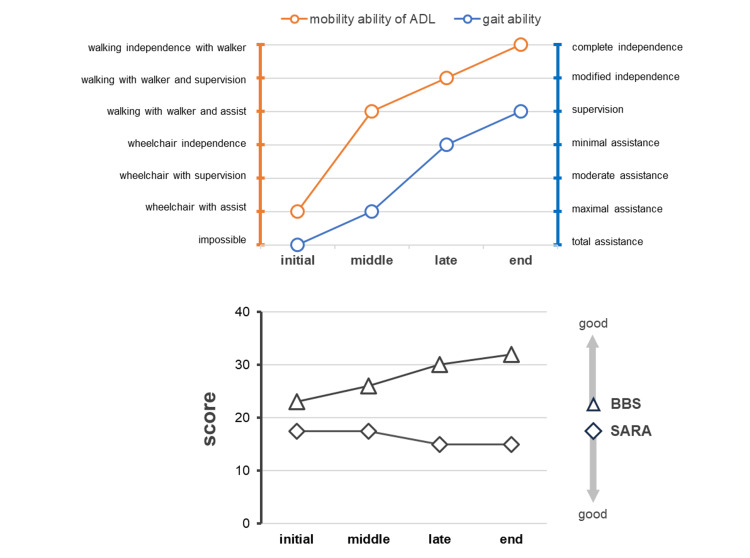
Progress of activities of daily living ADL and gait abilities, and BBS and SARA scores at four time points, from the initial to the end stage of the intervention. Initial, six weeks after fracture; middle, seven weeks after fracture; late, eight weeks after fracture; final, nine weeks after fracture. ADL: activities of daily living; BBS: Berg Balance Scale; SARA: Scale for the Assessment and Rating of Ataxia.

ADL ability, walking ability, BBS, and SARA scores at four time points from the beginning to the end of the intervention are shown in Figure 4. All items improved, and the patient was eventually discharged from the hospital with independent ambulation and gait monitoring. The SARA and BBS scores changed to 15/40 and 32/56, respectively, indicating an improvement compared to the baseline.

Progress of Standing Center of Gravity Sway Values

**Figure 5:**
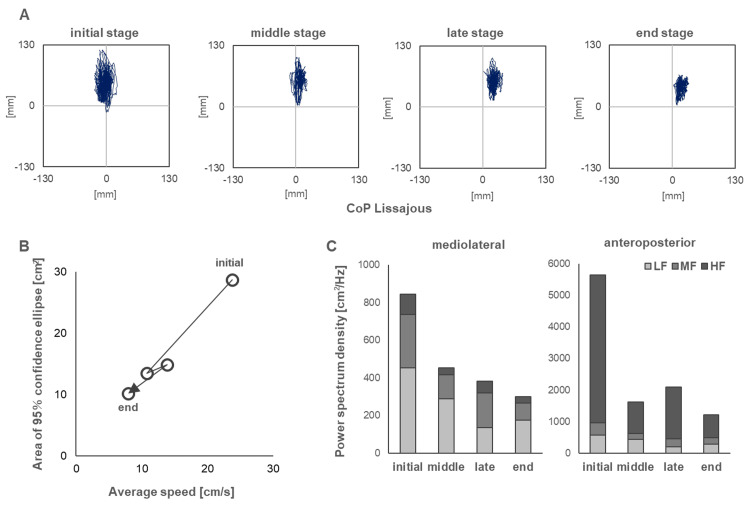
Progress of standing center of gravity sway values (A) The sway path of the center of gravity sways at four time points from the initial to the end stage of intervention. (B) The plot of sway area and sway speed for all stages. (C) The bar graph shows the average power values of LF, MF, and HF for all stages. Initial, six weeks after fracture; middle, seven weeks after fracture; late, eight weeks after fracture; final, nine weeks after fracture. LF: low frequency; MF: middle frequency, HF: high frequency.

Figure 5 shows the results of the center of gravity sway. Both the sway area and speed significantly decreased from the initial to the middle of the intervention period. The values tended to increase from the middle to the end of the study period but then began to decrease again from the end of the intervention period to the end of the study period. Figure 5A shows a large anterior-posterior sway and a gradual shift of the center of foot pressure position to the center. In contrast, the left and right centers of gravity progressively shifted to the right. Figure 5B shows the transition from the initial to the final stage, revealing that both the area and speed of movement decreased significantly from the initial to the middle stage. However, the average frequency value (Figure 5C) in the high-frequency band was particularly large in the initial back-and-forth directions. Subsequently, the power values in the high-frequency band decreased significantly from the beginning to the middle stage. The power values in the high-frequency band tended to increase slightly from the middle to the end of the period but decreased again from the end of the period to the end.

Progress of Lower Leg Muscle Activity

**Figure 6:**
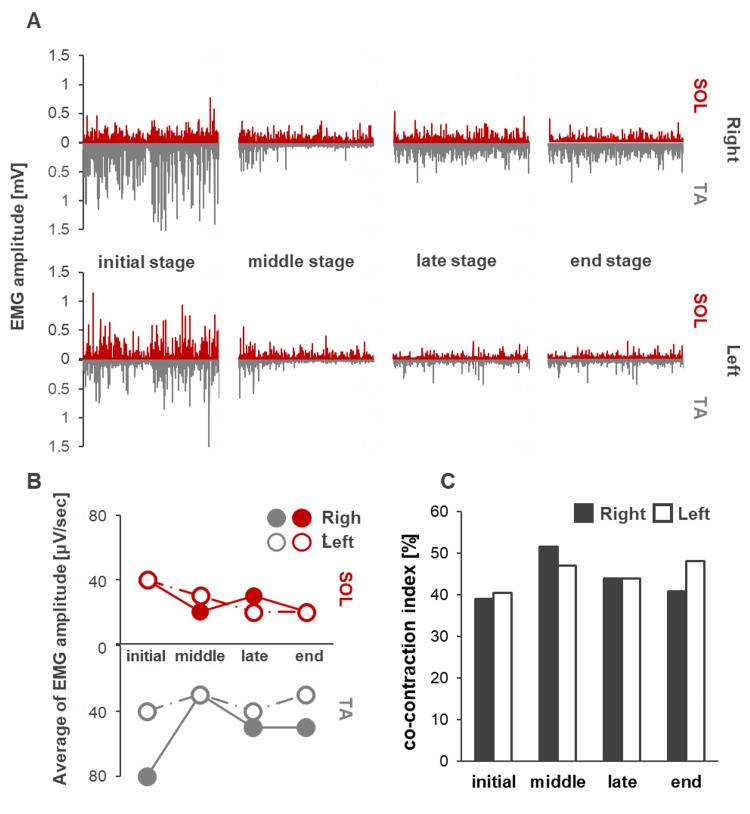
Progress of lower leg muscle activity (A) EMG waveforms of the TA and SOL muscles at four time points, from the initial to the end stage of intervention. (B) The line graph of the average of EMG amplitude of TA and SOL. Colored marks indicate right and white marks indicate left. (C) The bar graph shows the co-contraction index (%) for all stages. Colored bars indicate right and white bars indicate left. Initial, six weeks after fracture; middle, seven weeks after fracture; late, eight weeks after fracture; final, nine weeks after fracture. EMG: electromyography; TA: tibialis anterior; SOL: soleus.

The initial mean amplitude of the left TA activity was marked (Figure 6). Left TA activity decreased from initial to mid-term, and SOL activity decreased slowly from initial to mid-term on both the right and left sides (Figure 6B). In contrast, the CI increased from the initial to mid-term and decreased from the mid to late-term. In the final stage, the left TA-SOL index increased slightly. The detailed values of (2) and (3) are listed in Table 2. Compared with the age-matched healthy subject's data, the fluctuations and muscle activities were still large.

**Table 2:** Progress of standing center of gravity sway values and lower leg muscle activity values Initial, six weeks after fracture; middle, seven weeks after fracture; late, eight weeks after fracture; final, nine weeks after fracture. SCA31: spinocerebellar ataxia type 31; EMG: electromyography; LF: low frequency; MF: medium frequency; HF: high frequency.

		Patient with spinocerebellar degeneration SCA31				Healthy subject (n = 1)
		Initial	Middle	Late	End	
Area of 95％ confidence ellipse (cm^2^)		28.69	13.48	14.89	10.16	2.84
Average speed (cm/s)		23.76	10.75	13.83	7.94	1.36
Anteroposterior range of sway (cm)		13.07	10.04	9.1	6.37	3.55
Anteroposterior average position (cm)		5.09	5.86	5.58	4.5	3.38
Mediolateral range of sway (cm)		4.49	3.56	3.84	3.03	1.40
Mediolateral average position (cm)		-0.16	1.01	2.2	1.88	0.32
Power spectrum density (cm/Hz)						
Anteroposterior	LF	574.14	448.95	199.72	287.75	142.80
	MF	395.92	185.58	253.5	201.4	22.40
	HF	4668.52	986.12	1642.46	727.03	0.60
Mediolateral	LF	453.9	288.27	136.44	175.73	33.00
	MF	283.97	129.35	182.79	90.29	9.10
	HF	107.41	35.87	62.24	32.97	0.30
Average of EMG amplitude (μV/sec)						
Soleus	Right	40	20	30	20	4
	Left	40	30	20	20	16
Tibialis anterior	Right	80	30	50	50	5
	Left	40	30	40	30	10
Co-contraction index (%)						
	Right	39.01	51.52	43.92	40.90	27.88
	Left	40.51	46.96	44.00	48.09	61.18

Cross-Lagged Correlation Analysis

A significant correlation (r = -0.95, p < 0.038) was found between the left TA-SOL CI and SARA scores without a time difference (LAG0). However, no significant correlation was found between the CI and BBS (Table 3).

**Table 3:** Cross-lagged time-lag correlation analyses CI: co-contraction index; TA: tibialis anterior; SOL: soleus; SARA: Scale for the Assessment and Rating of Ataxia; BBS: Berg Balance Scale; * p < 0.05.

	SARA	BBS
The CI of the left TA-SOL muscles	r = -0.95 (LAG:0)*	r = 0.70 (LAG:0)
The CI of the right TA-SOL muscles	r = -0.32 (LAG:0)	r = -0.04 (LAG:0)

## Discussion

The patient was admitted to our hospital six years after the SCA diagnosis with bilateral lower extremity fractures because of a fall. After a six-week period for fracture treatment, the patient was allowed to stand and began to practice standing and walking during physical therapy. At that time, excessive effort to maintain standing postural balance was observed. Therefore, the strategy of lightly touching the back of the body (cervical region) to the wall surface was adopted, and static postural balance exercises were introduced to reduce such excessive effort. When the static postural balance was stabilized, we started dynamic postural balance exercises, such as tasks requiring voluntary center of gravity shifts and interarticular coordination exercises to control the upper and lower limbs while maintaining postural balance. Improvements in gait ability, the standing postural balance index (BBS), and the ataxia score (SARA) were observed as a result. In addition, interesting longitudinal results were obtained regarding the center of gravity sway and electromyographic findings in the standing posture. A discussion of the results is as follows.

Ability to maintain standing postural balance during the initial stage

The initial postural balance, swaying amplitude, area, speed, and power values of the high-frequency components were significantly high and characteristically higher than those of normal participants in the same age group. In particular, the increased sway amplitude in the anteroposterior direction and high frequency of the sway component were consistent with the characteristics of postural balance in patients with SCD previously reported [19,20]. Stabilizing standing postural balance requires the center of gravity to be positioned anteriorly to the ankle joint axis; the predictive activity of the triceps femoris muscle plays an important role in preventing the body from falling forward [21]. In static standing, control of strategies that increase ankle stiffness is crucial to maintaining the center of gravity at the center of the base plane of the support [22]. In cases of SCD, characterized by increased postural sway, maintaining the center of gravity within the basal plane of the support using a strategy of increased joint stiffness is common [23,24]. However, in the present case, the stiffness strategy was presumably not fully functional due to the overactivity of the right TA muscle observed in the early stages of intervention, which did not increase the time-synchronized CI. Moreover, the high frequency and speed of the initial sway component suggested that a fast sway was produced in this case, and the nature of such sway led us to consider the appearance of irregular and frequent movements of the ankle joint. We believe that this overcontraction of the right TA compensated for the ataxia of the left lower limb.

Ability to maintain standing postural balance from the initial to the mid-term period

Postural balance exercises with light touch were performed during physiotherapy to reduce postural sway during the early phase. One week later, in the mid-term period, not only the area but also the speed of swaying and power value in the high-frequency band decreased. Additionally, the activity of the right TA muscle was also significantly reduced. Because ataxia was also observed in the upper extremities in this case, light touch, which involves lightly touching the back of the body to the wall surface, was employed. The use of additional somatosensory information has been identified as an effective tool to reduce postural sway [15]. Thus, the reduction in sway area was considered to be an effect of the light touch. Furthermore, a reduction in velocity and high-frequency component of the sway was observed, suggesting that the rapid and erratic movements of the ankle joints decreased. Interestingly, an increase in the CI of the left and right TA-SOL was observed in the medium term. Previous reports are contradictory, with some affirming that the light touch effect decreases co-contraction of lower leg muscle activity along with reduction of the center of gravity sway [25,26], and others reporting that light touch to a fixed object increases co-contraction [27]. In the present case, an initial overcontraction of the right TA muscle occurred to compensate for the ataxia of the left lower extremity, resulting in a low CI with the SOL muscle. We believe that the reduction in sway due to the light touch effect decreased the overcontraction of the right TA muscle, which in turn stabilized the static standing postural balance from the initial to the intermediate period through a stiffness strategy involving co-contraction with the SOL muscle. In addition, cross-lagged correlation analysis showed a correlation between the CI of the left TA-SOL and SARA, with no time difference. Because this result was negatively correlated, we believe that ataxia was improved by co-contraction of the left lower extremity.

Ability to maintain postural balance from the midterm to the final period of intervention

The CI decreased from the mid to the end-term of the intervention. Co-contraction reportedly decreases when a subject is released from the threat of swaying [28], and is increased during effortful postural control, compared with the relaxed condition [12]. A decrease in co-contraction can be considered a decrease in effortful sway control and increased automaticity. When postural sway control becomes automatic, the sway velocity and the high-frequency band of sway have been reported to decrease [11]. These results are consistent with the progress observed in the present case. Furthermore, this progression is similar to findings in motor development and learning. In a previous study on healthy infants, the center of gravity sway was observed in three stages: (1) when the child needed assistance to maintain a sitting position; (2) when the child could maintain a sitting position for a short period; and (3) when the child could maintain a sitting position independently [29]. The results showed that the patient initially adopted a rigid strategy, freezing the degrees of freedom of movement to reduce sway. Once the postural balance is stabilized, the strategy shifts to releasing these degrees of freedom, allowing for free use of the upper limbs. However, this release of movement did not result in the previously observed co-contraction [30]. In this case, dynamic center of gravity transfer exercises using the upper extremities were actively performed during the late middle and final phases of the intervention. This differs from static postural control, which requires interjoint coordination. Even with this practice, no high-frequency increase in the sway velocity or frequency components was observed. Our results suggest that clinical procedures designed for motor learning are important, even in patients with SCA.

Limitations of this case study

This case study had several limitations. Gait training, which was initiated after mid-term, may have affected static postural control. Withdrawal from gait training is not realistic in physiotherapy practice and a prospective study with controlled interventions is required to investigate this effect. However, a meta-analysis of patients with stroke showed that although gait training is effective for dynamic balance, it has limited effects on static balance [31]. Therefore, the effect on static postural sway in the present case was unlikely to have been influenced by gait training. In the cross-lagged correlation analysis, an association was observed between a decrease in the CI and an improvement in SARA scores; however, no association was observed for the BBS. Because the BBS involves a more dynamic test, task specificity may be involved, such as when co-contraction is required or when the task cannot be completed with the appearance of co-contraction. As more cases are studied, examining the relationship between BBS sub-items and co-contraction should be possible. In the present study, a slight increase in the CI was observed at the final time point. According to the dynamical systems theory, postural muscle tone evolves in a series of increases and decreases that contribute to the ability to control posture and release degrees of freedom [32]. To clarify these causal relationships, we believe that an analysis of both cross-sectional and longitudinal data, including large sample size, is necessary.

## Conclusions

In conclusion, the intervention aimed at reducing postural sway with an initial light touch on the back improved postural balance and reduced ataxia symptoms in a patient with SCA31. Further enhancement with dynamic exercises for inter-articular coordination also contributed to these positive outcomes. Additional research is essential to consolidate these results and explore broader applications for SCA rehabilitation.
